# Bringing the Ca^2+^ sensitivity of myristoylated recoverin into the physiological range

**DOI:** 10.1098/rsob.200346

**Published:** 2021-01-06

**Authors:** Valerio Marino, Matteo Riva, Davide Zamboni, Karl-Wilhelm Koch, Daniele Dell'Orco

**Affiliations:** 1 Department of Neurosciences, Biomedicine and Movement Sciences, Section of Biological Chemistry, University of Verona, 37134 Verona, Italy; 2 Department of Neuroscience, Division of Biochemistry, University of Oldenburg, 26111 Oldenburg, Germany

**Keywords:** conformational selection, myristoyl switch, neuronal calcium sensor, phototransduction, rhodopsin kinase

## Abstract

The prototypical Ca^2+^-sensor protein recoverin (Rec) is thought to regulate the activity of rhodopsin kinase (GRK1) in photoreceptors by switching from a relaxed (R) disc membrane-bound conformation in the dark to a more compact, cytosol-diffusing tense (T) conformation upon cell illumination. However, the apparent affinity for Ca^2+^ of its physiologically relevant form (myristoylated recoverin) is almost two orders of magnitude too low to support this mechanism *in vivo*. In this work, we compared the individual and synergistic roles of the myristic moiety, the GRK1 target and the disc membrane in modulating the calcium sensitivity of Rec. We show that the sole presence of the target or the disc membrane alone are not sufficient to achieve a physiological response to changes in intracellular [Ca^2+^]. Instead, the simultaneous presence of GRK1 and membrane allows the T to R transition to occur in a physiological range of [Ca^2+^] with high cooperativity via a conformational selection mechanism that drives the structural transitions of Rec in the presence of multiple ligands. Our conclusions may apply to other sensory transduction systems involving protein complexes and biological membranes.

## 1. Background

Recoverin (Rec) is a 23 kDa neuronal calcium sensor (NCS) belonging to the EF-hand superfamily of Ca^2+^-binding proteins [1]. It is mostly expressed in vertebrate photoreceptors, where it contributes to the regulation of the phototransduction cascade by responding to the transient drop in the concentration of intracellular Ca^2+^ that follows the light-activation of rhodopsin [2,3]. Several lines of evidence suggest that Rec and its orthologues inhibit rhodopsin kinase (GRK1) at high Ca^2+^, in the dark state of the rod photoreceptor [4–7]. When [Ca^2+^] drops following illumination, such inhibition terminates and GRK1 becomes available to phosphorylate photoactivated rhodopsin (Rh*), a crucial step for achieving a timely shut-off of the visual cascade [2,8].

**Figure 1.**
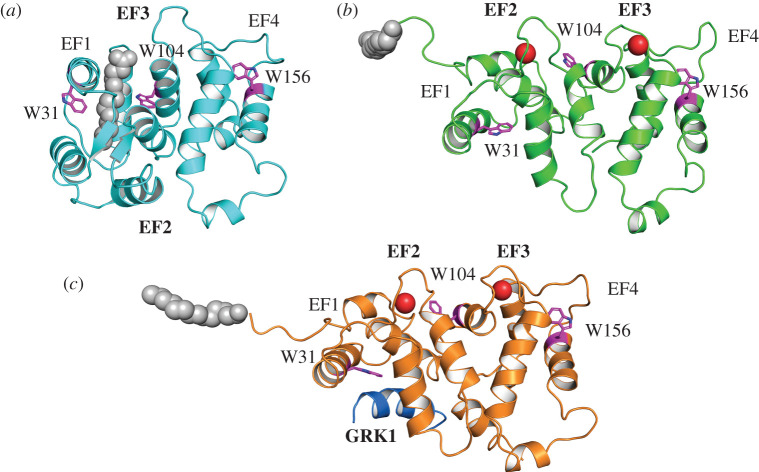
Three-dimensional structure of mRec in its (*a*) apo (PDB: 1IKU [11], (*b*) Ca^2+^-loaded (PDB: 1JSA [9]) and (*c*) Ca^2+^-loaded and GRK1-bound form (PDB: 2I94 [10]. Protein structure is shown as (*a*) cyan, (*b*) green and (*c*) orange cartoon, the myristoyl moiety is shown as grey spheres, Ca^2+^ ions are shown as red spheres, GRK1 peptide is displayed as blue cartoon and labelled in bold, residues W31, W104 and W156 are labelled and represented as purple sticks with N atoms highlighted in blue. EF1 to EF4 are labelled, Ca^2+^-binding EF2 and EF3 are labelled in bold.

NMR spectroscopy [9–11] and X-ray crystallography [12–15] have greatly contributed to our understanding of the structure-function characteristics of Rec highlighting the existence of a ‘tense' (T), compact conformation and a ‘relaxed' (R), more elongated conformation, in which the four EF-hands motifs in Rec acquire different relative orientations (figure 1). Since EF1 and EF4 are non-functional EF-hands [13] Rec binds only two Ca^2+^ ions in the EF2 (low-affinity site) and EF3 (high-affinity site) motifs located, respectively, in the N and in the C terminal domains [16,17]. Acylation of N-terminus of Rec, most often a myristoylation [18] is essential for the so-called myristoyl-switch mechanism following Ca^2+^ binding. The myristoyl moiety is sequestered into a hydrophobic groove in the T state (figure 1*a*), while it becomes solvent-exposed upon Ca^2+^ binding, when the transition to the R state occurs (figure 1*b*) [9,11,19]. This switch causes the exposure of hydrophobic residues on the protein surface, which allows the binding of the N-terminal region of GRK1 (residues 1–25; figure 1*c*) [10,20] and provides an anchor for binding to the surface of rod outer segment (ROS) disc membranes [21], thus forming a complex where Rec-GRK1 is in close proximity to Rh without inducing its phosphorylation. When [Ca^2+^] decreases, the myristoyl group returns in its binding pocket, thus dissociating the GRK1 N-terminal stretch and causing Rec to switch to the more soluble T state.

Calcium binding occurs via a sequential, non-cooperative mechanism in non-myristoylated Rec (nmRec) with moderate to high affinity for the specific binding sites (*K*_D_^EF2^ = 6.9 µM; *K*_D_^EF3^ = 0.11 µM) [16]. By contrast, the Ca^2+^ binding to myristoylated recoverin (mRec) is highly cooperative and has been successfully described by a concerted allosteric model [16], in which multiple equilibria exist between R and T states, each conformation being possibly populated at different occupation levels of the Ca^2+^ binding sites. The myristoyl group is thought to act as an allosteric inhibitor that keeps mRec in the T state in aqueous solution and decreases its apparent affinity for Ca^2+^ [16,22] (*K*_D_^app^ = 17–18 µM). In opposition, nmRec exists predominantly in the R state and the sequential, non-cooperative binding reflects the intrinsic affinities of the two EF-hands for Ca^2+^ ions [16]. It is plausible that changes in the chemical environment may affect the stability of the T state and could thus cause the equilibrium to shift towards the R state even without changes in the intrinsic Ca^2+^ binding constants. For example, mutations in mRec that decrease the hydrophobic interactions between the myristoyl moiety and its binding pocket enhance two to fourfold the apparent affinity of mRec for Ca^2+^ [22] and shift the equilibrium from the T state towards the R state, without affecting the intrinsic binding constants to EF2 and EF3 [22].

The occurrence of the T to R conformational transition is a crucial requirement for the *in vivo* function of mRec, as the extrusion of the myristoyl group is necessary to keep mRec anchored to the ROS membrane to inhibit GRK1. On the other hand, prompt sequestration of the myristoyl group in the protein milieu at low [Ca^2+^] is essential to permit GRK1 function. While both experimental studies [21,23] and molecular dynamics simulations [24,25] are consistent with the notion that Ca^2+^-bound mRec stabilizes the R state and allows the spontaneous insertion of the myristic moiety into the ROS disc membrane, the low apparent affinity (*K*_D_^app^ = 17–18 µM) for Ca^2+^ binding of mRec in aqueous solution raised concerns as to the actual role of Rec *in vivo* [26–28], where [Ca^2+^] varies in a narrow sub-micromolar range (20–250 nM in mouse rods [29], but see ref. [30] for higher values in other vertebrates). It has been suggested that ROS membranes [16] and/or the presence of the GRK1 target [22] could favour the T to R transition and shift it into the physiological range of intracellular [Ca^2+^], but so far experimental evidence for either hypothesis was missing.

Here we used absorption, fluorescence and circular dichroism spectroscopy to show that neither the sole presence of the GRK1 target nor the ROS membrane alone are sufficient to achieve a physiological response of mRec to changes in intracellular [Ca^2+^], although they individually decrease the half maximal effective concentration of [Ca^2+^] necessary to complete the transition to the R state (EC_50_). Interestingly, the simultaneous presence of GRK1 and membrane allows the T to R transition of mRec to occur in a physiological range of [Ca^2+^] with high cooperativity.

## 2. Results

### 2.1. Effects of the GRK1 target on the Ca^2+^ affinity of Rec variants monitored by competition with chromophoric chelators

To evaluate the effect of the presence of GRK1 peptide on the Ca^2+^ affinity of Rec variants, we employed two different techniques based on the competition with Ca^2+^ chelators with different Ca^2+^ affinity and peculiar physico-chemical properties, namely Br_2_-BAPTA (Ca^2+^ affinity = 2.3 µM) and Oregon Green-conjugated BAPTA-5N (OG BAPTA-5N, Ca^2+^ affinity = approx. 20 µM) [31].

**Figure 2.**
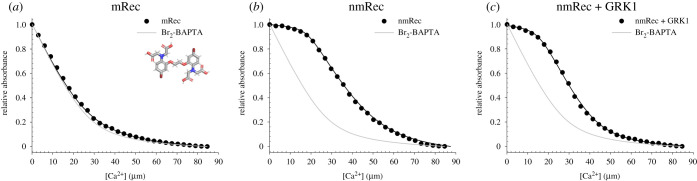
Example of Ca^2+^ titrations in competition with the chromophoric chelator 5,5′Br_2_-BAPTA. (*a*) Ca^2+^ titrations of 28 µM mRec (black) and 28 µM 5,5′Br_2_-BAPTA. Inset shows the molecular structure of the chromophoric chelator 5,5′Br_2_-BAPTA. (*b*) Ca^2+^ titration of 28 µM nmRec (black) and 28 µM 5,5′Br_2_-BAPTA. (*c*) Ca^2+^ titration of 28 µM nmRec (black), 42 µM GRK1 peptide and 28 µM 5,5′Br_2_-BAPTA. Experimental points are shown together with the optimal curve (black line) calculated by computer fitting and the theoretical curve representing a titration in the sole presence of 5,5′Br_2_-BAPTA (grey line). Estimation of the Ca^2+^-binding constants is reported in table 1, data normalization is detailed in the Methods section.

Due to its low apparent Ca^2+^ affinity (approx. 17 µM) [16,32], mRec was not able to compete for Ca^2+^ with Br_2_-BAPTA (figure 2*a*), exhibiting a titration curve almost indistinguishable from the theoretical curve of the chelator alone. On the other hand, Ca^2+^ titration of nmRec (figure 2*b*) allowed the estimation of the macroscopic binding constants for both functional EF-hands, resulting in *K*_d_^EF3^ = 43 nM and *K*_d_^EF2^ = 12.4 µM (table 1), in line with previously published results [16]. Interestingly, as previously shown by other Ca^2+^-sensor proteins such as Calmodulin [33], nmRec displayed a significant increase in Ca^2+^ affinity in the presence of the target GRK1 (figure 2*c*). Specifically, while EF3 was substantially unaffected by GRK1 in terms of Ca^2+^ affinity (*K*_d_^EF3^ = 44 nM, table 1), a 100-fold increase was displayed by EF2, whose affinity increased from 12.4 µM to 0.12 µM (table 1).

**Table 1.** Macroscopic Ca^2+^-binding constants of Rec variants in the absence and in the presence of the target GRK1.

variant	logK_a_^EF3^	K_d_^EF3^	logK_a_^EF2^	K_d_^EF2^
nmRec^a^	7.37 ± 0.12	43 nM	4.91 ± 0.12	12.4 µM
nmRec + GRK1^a^	7.35 ± 0.11	44 nM	6.92 ± 0.09	0.12 µM
mRec^b^	4.75 ± 0.13	18.0 µM		
mRec + GRK1^b^	4.91 ± 0.09	12.2 µM		

^a^Estimated by competition with Br_2_-BAPTA.

^b^Estimated by competition with OG BAPTA-5N.

**Figure 3.**
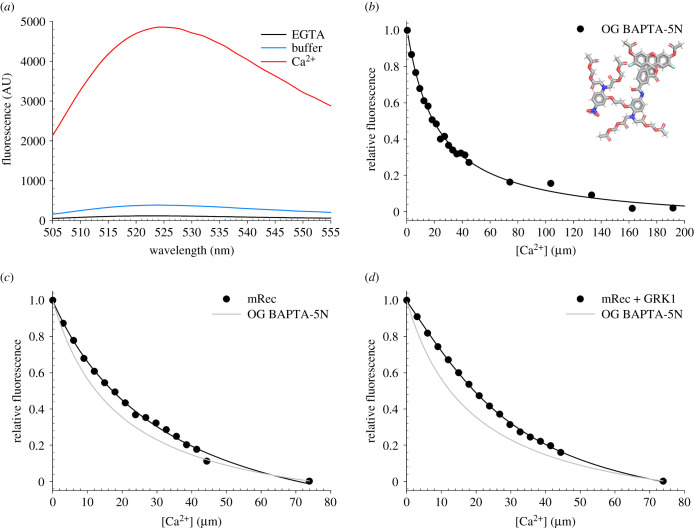
Example of Ca^2+^ titrations in competition with the fluorescent chelator Oregon Green (OG) BAPTA-5N. (*a*) Fluorescence emission spectra of 0.5 µM OG BAPTA-5N after excitation at 494 nm in decalcified buffer (blue) and after sequential additions of 200 µM EGTA (black) and 600 µM Ca^2+^. (*b*) Ca^2+^ titrations of 0.5 µM OG BAPTA-5N. Inset shows the molecular structure of the fluorescent chelator OG BAPTA-5N. (*c*) Ca^2+^ titrations of 15 µM mRec (black) and 0.5 µM OG BAPTA-5N. (*d*) Ca^2+^ titrations of 15 µM mRec (black), 22.5 µM GRK1 peptide and 0.5 µM OG BAPTA-5N. Experimental points are shown together with the optimal curve (black line) calculated by computer fitting and the theoretical curve representing a titration of the sole OG BAPTA-5N (grey line). Estimation of the Ca^2+^-binding constants is reported in table 1, data normalization is detailed in the Methods section.

As no competition could be detected between mRec and Br_2_-BAPTA due to the substantially different affinities for Ca^2+^, we performed a competition assay by monitoring Ca^2+^-dependent fluorescence of the low-affinity fluorescent chelator OG BAPTA-5N (figure 3*a*). The high signal-to-noise ratio attributed to the intense fluorescence of the chelator excited at 494 nm allowed a precise estimation of initial [Ca^2+^] in the decalcified buffer and showed complete saturation at approximately 200 µM [Ca^2+^] (figure 3*a*). Under the tested experimental conditions, the detected affinity of OG BAPTA-5N for Ca^2+^ (log *K*_a_ = 4.73 ± 0.06, corresponding to a *K*_d_ = 18.6 µM; figure 3*b*) was suitable for competition assays with Rec, therefore we performed Ca^2+^ titrations of mRec both in the absence and in the presence of GRK1 target. Data fitting to a one-site model for mRec (figure 3*c*) estimated a *K*_d_ of 18.0 µM (table 1), identical to that reported in the literature [16], which decreased to 12.2 µM (table 1) in the presence of the GRK1 peptide (figure 3*d*), displaying a similar behaviour to that of nmRec, although to a lesser extent. No fitting to a two-site model was possible in this case.

### 2.2. Effects of the GRK1 target on the Ca^2+^-dependent conformational transition of mRec monitored by FRET analysis

**Figure 4.**
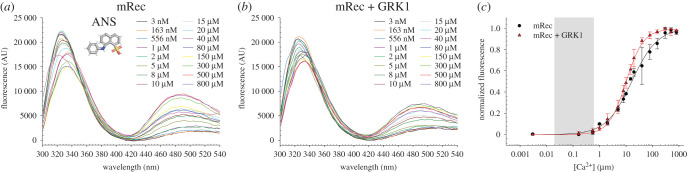
Fluorescence spectra of 2 µM mRec in the presence of (*a*) 25 µM ANS and (*b*) 25 µM ANS and 3 µM GRK1 peptide at increasing Ca^2+^ concentrations (3 nM black, 163 nM orange, 556 nM blue, 1 µM violet, 2 µM green, 5 µM olive, 8 µM dark green, 10 µM dark red, 15 µM light grey, 20 µM cyan, 40 µM magenta, 80 µM dark blue, 150 µM yellow, 300 µM light green, 500 µM red, 800 µM purple). Spectra were recorded in the 300–540 nm range upon excitation at 280 nm. Inset in (*a*) shows the molecular structure of the hydrophobic probe ANS. (*c*) Dependence of the FRET signal on Ca^2+^ titrations of mRec (black) and mRec + GRK1 peptide (red) monitored by the ratio between the emission peak of intrinsic fluorescence and ANS fluorescence. Data refer to the average ± standard deviation of three independent runs and are shown together with the optimal fitting to a three-parameter Hill sigmoid and the physiological Ca^2+^ concentration in ROS (20 nM–600 nM) in light grey. Estimation of the EC_50_ is reported in table 2, data normalization is described in detail in Material and methods section.

Myristoylated Rec is known to be subjected to a conformational change upon Ca^2+^ binding called myristoyl-switch, which results in a substantial increase in exposure of hydrophobic surface, mainly due to the externalization of aromatic residues, in particular of W31, W104 and W156 (figure 1). Therefore, by exploiting the FRET between the intrinsic fluorescence emission and the emission of the hydrophobic probe ANS at increasing Ca^2+^ concentration, we estimated the concentration at which half of the mRec pool undergoes the T to R transition (EC_50_) in the absence (figure 4*a*) and in the presence of GRK1 peptide (figure 4*b*). Specifically, we monitored the ratio between the emission peak of the intrinsic fluorescence and the emission peak of ANS. Results summarized in figure 4*c* highlight a minor but significant decrease in EC_50_ exhibited by mRec in the presence of the target (16.64 ± 1.60 µM versus 10.07 ± 0.33 µM, table 2), in line with the Ca^2+^ affinity variations. Such decrease in EC_50_ is accompanied by an increase of the Hill coefficient from 0.90 ± 0.07 to 1.32 ± 0.06, suggesting an enhanced cooperativity of the structural transition from T to R.

**Table 2.** Biophysical analysis of the structural transition of Rec variants in the absence and in the presence of the GRK1 peptide and LP.

variant	EC_50_ (µM)	Hill coefficient
nmRec^a^	(156.8 ± 37.3) × 10^−3^	0.57 ± 0.09
nmRec + GRK1^a^	(44.9 ± 2.3) × 10^−3^	1.32 ± 0.09
mRec^a^	25.12 ± 7.28	0.63 ± 0.07
mRec + GRK1^a^	9.44 ± 1.90	0.63 ± 0.06
mRec^b^	16.64 ± 1.60	0.90 ± 0.07
mRec + GRK1^b^	10.07 ± 0.33	1.32 ± 0.06
mRec + LP^a^	0.37 ± 0.68	0.30 ± 0.14
mRec + GRK1 + LP^a^	0.13 ± 0.02	2.15 ± 0.79

^a^Estimated by CD spectroscopy.

^b^Estimated by intrinsic fluorescence-ANS FRET.

### 2.3. Effects of the GRK1 target on the Ca^2+^-dependent conformational transition of Rec variants monitored by CD spectroscopy

CD spectroscopy offers the unique advantage to allow the direct monitoring of protein secondary and tertiary structure in solution under conditions that mimic the physiological ones. Such structural changes can be directly monitored in the far and near UV spectral range, respectively, and experiments can be performed in the co-presence of ligands. We thus used CD spectroscopy to directly probe the effect of the myristoyl moiety and the GRK1 peptide on Rec conformation at different Ca^2+^ levels.

**Figure 5.**
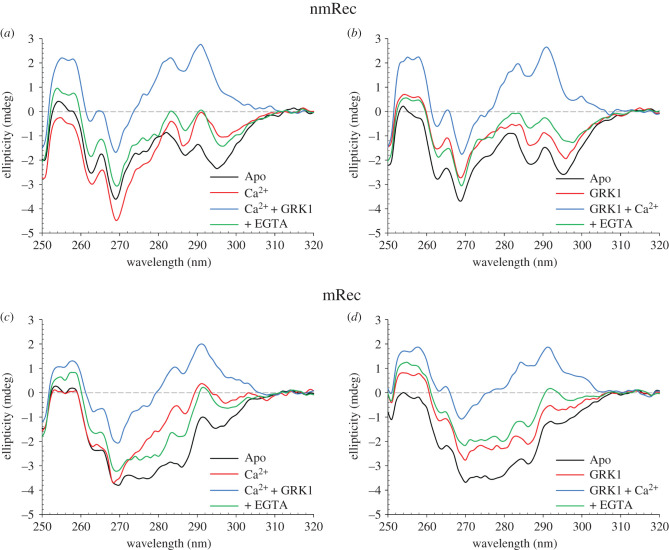
(*a*) Near UV CD spectra of 40 µM nmRec and 300 µM EGTA (black), after sequential additions of 1 mM free Ca^2+^ (red), 60 µM GRK1 peptide (blue) and 4.2 mM free EGTA (green). (*b*) Near UV CD spectra of 40 µM nmRec and 300 µM EGTA (black), after sequential additions of 60 µM GRK1 peptide (red), 1 mM free Ca^2+^ (blue) and 4.2 mM free EGTA (green). (*c*) Near UV CD spectra of 40 µM mRec and 300 µM EGTA (black), after sequential additions of 1 mM free Ca^2+^ (red), 60 µM GRK1 peptide (blue) and 4.2 mM free EGTA (green). (*d*) Near UV CD spectra of 40 µM mRec and 300 µM EGTA (black), after sequential additions of 60 µM GRK1 peptide (red), 1 mM free Ca^2+^ (blue) and 4.2 mM free EGTA (green).

Near UV CD spectra of nmRec showed a relatively small conformational change upon Ca^2+^ binding (figure 5*a*, black and red lines), in line with previous results [23,34] and with the notion that, even in the absence of Ca^2+^, nmRec is sampling the R state. Binding of Ca^2+^-loaded nmRec to GRK1 resulted in a major rearrangement of the microenvironment of all three aromatic residues as shown by the significant positive spectral shift (figure 5*a*, blue line), especially apparent in the Phe and Trp bands. On the contrary, addition of EGTA brought the free [Ca^2+^] to 120 nM and caused a decrease in ellipticity in the Phe band and a sign inversion of the signal in the Tyr and Trp bands, indicative of a substantial structural variation towards a conformation different from the apo form in the absence of the target (figure 5*a*, green line, compared to black line). Interestingly, the inversion in the order of ligand additions (figure 5*b*) showed differences between apo nmRec in the absence and in the presence of the GRK1 peptide in all spectral bands, suggesting an interaction even in the absence of Ca^2+^ (figure 5*b*, black and red lines). Further addition of Ca^2+^ induced the same Ca^2+^-loaded and GRK1-bound conformation as in figure 5*a* (compare with figure 5*b*, blue lines). Chelation of Ca^2+^ upon addition of EGTA again resulted in a negative shift of the spectrum to a conformation similar to that of the apo form in the presence of the GRK1 peptide, with small differences in the Trp band, probably due to the slightly higher free Ca^2+^ concentration under this condition.

Similarly to nmRec, also mRec displayed its typical change in the fingerprint of the protein tertiary structure upon Ca^2+^ binding (figure 5*c*). Addition of the GRK1 peptide to Ca^2+^-bound mRec resulted in a positive shift involving all three spectral regions (blue lines), very similar to the relative cases shown in figure 5*a* and *c*. Moreover, Ca^2+^ chelation by EGTA triggered the transition to a different conformation, suggesting again a potential interaction between apo mRec and the GRK1 peptide. Such interaction was confirmed by inverting the addition of the interactors, as the presence of GRK1 caused a positive spectral shift especially in the Phe and Tyr bands (figure 5*d*, red line). Addition of Ca^2+^ resulted in a further positive shift with a fine structure comparable to that exhibited in figure 5*c* and in the case of nmRec (compare with figure 5*a–c*, blue lines). Finally, addition of EGTA caused a negative shift, reverting the fine structure of spectrum to that of the apo GRK1-bound form, with small differences in intensity attributable to the residual free Ca^2+^.

As far as the compactness and the secondary structure of the Rec variants are concerned, far UV CD spectra were substantially in line with the behaviour exhibited in the near UV. Indeed, nmRec displayed an increase in α-helix content and compactness upon Ca^2+^ binding (electronic supplementary material, figure S1A) accompanied by an increase of the *θ*_222_/*θ*_208_ ratio from 0.84 to 0.88 (table 3), which indicates an overall reshaping of the spectrum. The addition of the GRK1 peptide resulted in a substantial increase in ellipticity due to the α-helical folding of the peptide and a corresponding variation of the spectral shape (*θ*_222_/*θ*_208_ = 0.94, table 3). Removal of Ca^2+^ by EGTA decreased the signal and induced a variation of the shape (*θ*_222_/*θ*_208_ = 0.87), suggesting again a mild interaction of the GRK1 peptide with nmRec even in the absence of Ca^2+^. In line with the near UV CD spectra, also far UV CD spectra displayed a small but significant spectral variation upon addition of the GRK1 peptide in the absence of Ca^2+^ (electronic supplementary material, figure S1B), both in terms of intensity and shape (*θ*_222_/*θ*_208_ = 0.84 versus 0.86, table 3). Ca^2+^ addition again caused a major increase in compactness shown by the more negative spectrum as well as by the *θ*_222_/*θ*_208_, reaching 0.94 (table 3), while addition of EGTA resulted in a drop of both ellipticity and *θ*_222_/*θ*_208_ ratio (0.87). A similar behaviour was displayed by mRec under the same conditions, albeit with numerical differences. Binding of Ca^2+^ induced an ellipticity rise at 208 nm (electronic supplementary material, figure S1C) yielding a shift of the *θ*_222_/*θ*_208_ ratio from 0.81 to 0.91 (table 3), while the concomitant presence of GRK1 resulted in a noticeable enhancement of the spectrum and a further increase of the spectral descriptor (0.95, table 3). Also in this case, the addition of EGTA not only lowered the ellipticity (electronic supplementary material, figure S1C, green line), but it also altered the shape of the spectrum, resulting in a *θ*_222_/*θ*_208_ of 0.85, identical to that exhibited by apo mRec upon addition of the GRK1 peptide (electronic supplementary material, figure S1D, red line), pointing towards an interaction between mRec and its target even in the absence of Ca^2+^. Analogously to nmRec, the order of addition of the two interactors Ca^2+^ and GRK1 peptide did not matter, as the addition of Ca^2+^ enhanced both the intensity of the CD signal and the *θ*_222_/*θ*_208_ ratio to 0.95 (table 3), which was completely reversed upon EGTA addition (*θ*_222_/*θ*_208_ = 0.85).

**Table 3.** Spectral shape descriptors (*θ*_222_/*θ*_208_) of Rec variants upon sequential additions of Ca^2+^, GRK1 peptide and EGTA obtained by circular dichroism spectroscopy.

	Apo	+ Ca^2+^	+ GRK1	+ EGTA	Apo	+ GRK1	+ Ca^2+^	+ EGTA
mRec	0.81	0.91	0.95	0.85	0.81	0.85	0.95	0.85
nmRec	0.84	0.88	0.94	0.87	0.84	0.86	0.94	0.87

**Figure 6.**
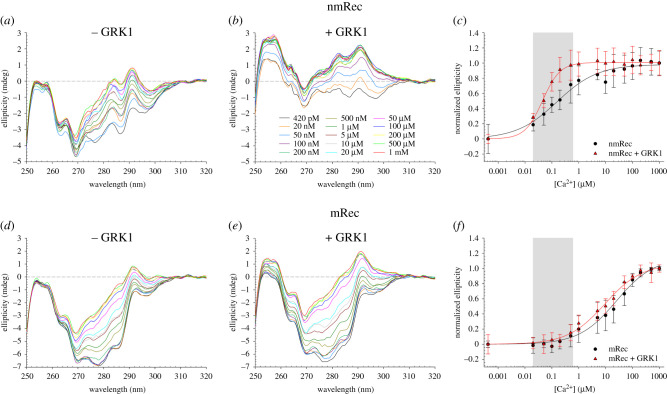
Near UV CD spectra of 43 µM nmRec (*a*) in the absence and (*b*) in the presence of 65 µM GRK1 peptide at increasing Ca^2+^ concentrations (420 pM black, 20 nM orange, 50 nM blue, 100 nM violet, 200 nM green, 500 nM olive, 1 µM dark green, 5 µM dark red, 10 µM light grey, 20 µM cyan, 50 µM magenta, 100 µM dark blue, 200 µM yellow, 500 µM light green, 1 mM red). (*c*) Ca^2+^ titrations of nmRec (black) and nmRec + GRK1 peptide (red) monitored by following the ellipticity at 292 nm. Near UV CD spectra of 43 µM mRec (*d*) in the absence and (*e*) in the presence of 65 µM GRK1 peptide at increasing Ca^2+^ concentrations. (*f*) Ca^2+^ titrations of mRec (black) and mRec + GRK1 peptide (red) monitored by following the ellipticity at 279 nm. Data refer to the average ± standard deviation of three independent replicas and are shown together with the optimal fitting to a three-parameter Hill sigmoid and the physiological Ca^2+^ concentration (20–600 nM) in light grey. Estimation of the EC_50_ is reported in table 2, data normalization is described in detail in Methods section.

To further investigate the Ca^2+^-dependent structural changes occurring in Rec variants and the effects exerted by the target peptide, we evaluated the evolution patterns of the near UV spectra at increasing Ca^2+^ concentration. Interestingly, results for nmRec highlighted a completely different evolution of the spectra upon Ca^2+^ titration between GRK1-free (figure 6*a*) and GRK1-bound (figure 6*b*) forms, which started and ended with substantially different conformations, suggesting an interaction between nmRec and GRK1 also in the absence of Ca^2+^. By monitoring the dichroism signal at 292 nm as a function of the Ca^2+^ concentration (figure 6*c*), we could estimate the concentration at which the conformational transition was half-maximal by means of fitting to a three-parameter Hill sigmoid. Results revealed not only a 3.5-fold decrease of the EC_50_ from 156.8 ± 37.3 nM to 44.9 ± 2.3 nM (table 2) in the presence of GRK1, but also a significant increase of the Hill coefficient from 0.57 ± 0.09 to 1.32 ± 0.09 (table 2) due to the target, indicative of a more cooperative structural transition. Overall, in line with Ca^2+^ affinity measurements, the EC_50_ of nmRec falls inside the physiological Ca^2+^ window in ROS (20–600 nM) regardless of the presence of the target. On the other hand, mRec exhibited larger variation in the Phe and Tyr spectral bands upon Ca^2+^ binding in the presence of the target compared to the isolated protein (figure 6*d* and *e*); nevertheless, the overall spectral shape features were not substantially perturbed. The evaluation of the ellipticity signal at 279 nm (figure 6*c*) after fitting to a three-parameter Hill sigmoid pointed to a decrease of the EC_50_ for mRec from 25.12 ± 7.28 µM to 9.44 ± 1.90 µM (table 2) in the presence of the GRK1, with no effects on the cooperativity of the process, as shown by the identical Hill coefficient (0.63, table 2). Notably, although the approximately 2.6-fold decrease of EC_50_ due to the target was comparable with that recorded with fluorescence titrations, at odds with nmRec mRec is unable to undergo its conformational change in the physiological Ca^2+^ range (20–600 nM) even in the presence of GRK1 (see the grey-shaded regions in figure 6*c* and *f*).

### 2.4. Effects of the ROS membrane on the Ca^2+^-dependent conformational transition of Rec variants monitored by CD spectroscopy

While the presence of the GRK1 peptide increased the apparent Ca^2+^ affinity and decreased EC_50_ of mRec, such values fell out of the physiological concentration of Ca^2+^ operating in photoreceptor ROS, therefore, we tested whether the presence of membranes would be able to further decrease the EC_50_.

To mimic *in vitro* the presence of membranes, we employed liposomes (LP) with the same composition as the ROS membranes. To ensure the stoichiometric excess of theoretical binding sites for mRec on LP, we measured the hydrodynamic diameter and the concentration of the vesicles by means of dynamic light scattering and nanoparticle tracking analysis (electronic supplementary material, figure S2), resulting in 75.3 ± 1.3 nm diameter LP and estimated the maximum number of mRec proteins potentially bound to LP (approx. 1400) as elucidated in the Methods section.

Near UV CD spectra of Ca^2+^-free mRec in the presence of LP showed small but significant differences in the Phe band (electronic supplementary material, figure S3) between GRK1-free and GRK1 bound, although the overall spectra were similar. It is noteworthy that even a low concentration (5 nM) of LP caused prominent signal scattering in the near UV; therefore, spectra were noisy due to the considerably lower concentration of mRec (7 µM) compared to experiments without LP (43 µM). Nevertheless, the experimental conditions ensured a reasonable signal-to-noise ratio.

**Figure 7.**
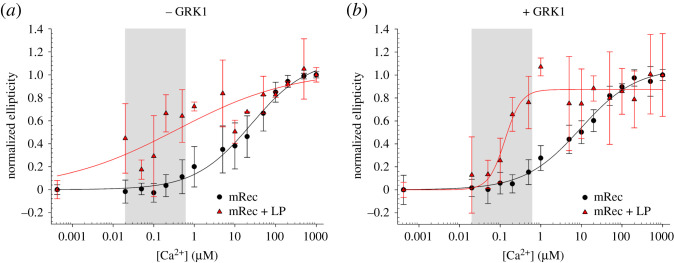
(*a*) Ca^2+^ titrations of 7 µM mRec in the absence (black) and in the presence of 5 nM LP (red) monitored by following the ellipticity at 279 nm. (*b*) Ca^2+^ titrations of 7 µM mRec and 10.5 µM GRK1 peptide in the absence (black) and in the presence of 5 nM LP (red) monitored by following the ellipticity at 279 nm. Data refer to the average ± standard deviation of three independent runs and are shown together with the optimal fitting to a three-parameter Hill sigmoid and the physiological Ca^2+^ concentration (20 nM–600 nM) in light grey. Estimation of the EC_50_ is reported in table 2, data normalization is described in detail in Methods section.

Again, by monitoring the ellipticity at 279 nm, we estimated the EC_50_ of mRec in the presence of LP, resulting in 0.37 ± 0.68 µM (table 2) with a Hill coefficient of 0.30 ± 0.14, 67-fold smaller than that exhibited in the absence of LP (figure 7*a*). Noticeably, the same experiment performed in the presence of GRK1 (figure 7*b*) resulted in a threefold decrease in EC_50_ to 0.13 ± 0.02 µM (table 2), 72-fold smaller than the EC_50_ estimated with no membranes. Moreover, the increase in Hill coefficient (2.15 ± 0.79) suggested a highly cooperative conformational change in mRec induced by the proximity of membranes and by the presence of the peptide target. Overall, near UV CD titrations showed that ROS membranes increase the affinity of mRec for Ca^2+^ to a larger extent with respect to the sole presence of the target GRK1 (EC_50_ = 0.37 ± 0.68 µM versus 9.44 ± 1.90 µM; table 2), nevertheless, only the concomitant presence of both membrane and target would allow mRec to respond to the decrease in Ca^2+^ concentration within the physiological Ca^2+^ window (EC_50_ = 0.13 ± 0.02 µM); figure 7*b*, grey shaded area).

## 3. Discussion

Several *in vitro* studies reported that the half-maximal inhibition of GRK1 by Rec occurs between 1.5 and 6 µM free Ca^2+^ [6,12,35], which is an order of magnitude higher than the physiological range of cytoplasmic Ca^2+^. This apparent discrepancy was theoretically solved by extrapolating the data to the *in vivo* conditions, namely by adjusting the membrane or rhodopsin concentration to the physiological value [6]. Furthermore, it was postulated that either the GRK1 target [22] or ROS membrane [16] could facilitate the T to R transition even in the absence of Ca^2+^and could then shift the Ca^2+^ sensitivity of mRec over the physiological range. No experimental tests of these hypotheses to reconcile experimental data with the physiological framework existed before our study, except one approach that showed Rec and calmodulin acting synergistically on GRK1, which can affect the Ca^2+^ sensitivity [35].

Another problem exists for the functional impact of the myristoyl group in Rec. While introducing cooperativity of Ca^2+^ binding, it makes the T to R transition incompatible with the physiological Ca^2+^ range. Ames *et al*. [16] pointed out that the exposed myristoyl group in mRec is thermodynamically unfavourable causing a lower Ca^2+^ affinity.

In the present work, we investigated the mutual influence of these parameters on the structural and functional aspects in Ca^2+^ signalling in photoreceptors. Our present data (figures 2*c* and 6*c*) indeed confirm that nmRec would be able to switch between Ca^2+^-free and Ca^2+^-bound conformations with EC_50_ values that are compatible with the physiological [Ca^2+^] changes upon rod illumination [16] and that the GRK1 target would bring this transition perfectly within this range (figure 6*c*). On the other hand, in line with the previous hypotheses we show that the target increases the apparent affinity of mRec for Ca^2+^ (figure 3) and decreases the corresponding EC_50_ (figures 4 and 6*f*), but such decrease is not sufficient for bringing the conformational changes to the physiological range. Membranes mimicking the disc composition influence the Ca^2+^ sensitivity of the conformational transition in mRec significantly (table 2), as the EC_50_ value decreases from 25.12 µM to 0.37 µM (mRec + LP, table 2). The EC_50_ of 0.37 µM is close to the physiological cytoplasmic Ca^2+^ range in mice rods and within the range found in lower vertebrates (see introduction), but the conformational switch lacks the cooperativity typically observed for Ca^2+^ binding to mRec [16,17,19]. Only the co-presence of the GRK1 peptide and LP shifted the EC_50_ exactly within the physiological [Ca^2+^] window (0.13 µM) and allowed the conformational transition to occur with high cooperativity (Hill coefficient = 2.15).

Molecular recognition in protein-ligand and protein–protein interactions is regulated by complex energetics often involving conformational changes in the macromolecules besides the establishment of specific bonds. Whether conformational changes are caused in response to ligand binding according to the induced fit model [36] or happen beforehand to produce a binding-competent state, according to the conformational selection model [37], is a highly debated subject with many examples existing in support of both scenarios. Recent lines of evidence show that, under Ca^2+^-saturating conditions, the binding between Rec and GRK1 is driven by conformational selection rather than induced fit [38]. It is tempting to assume that conformational selection might prevail also when considering multiple equilibria, in which the ligands of the same macromolecule (Rec) are manifold: Ca^2+^ ions, GRK1 target, myristoyl group and disc membrane, where the modified fatty acid can spontaneously insert. A formal description of the conformational selection mechanism is particularly challenging in the case of multiple equilibria as it would require both specific structural information on each state and complete knowledge on the kinetics of reaction pathways [39,40]. Although a rigorous description of the mRec's switch between T and R states in the co-presence of the GRK1 target and the membrane milieu is therefore extremely complex and out of the scope of this work, our data permit to infer some interesting consideration as to the prevalence of conformational selection also in the presence of multiple ligands. Figure 5 shows that the conformational transition from T to R is not necessarily induced by Ca^2+^ binding, as the interaction between the GRK1 peptide and both nmRec and mRec was observed prior to Ca^2+^ binding. While this is not surprising for nmRec, which is already in the R state, this implies that the GRK1 peptide, *per se* unstructured in solution [10] is capable to bind to mRec in the T state, where the myristoyl moiety is sequestered in the hydrophobic binding pocket that accommodates the GRK1 stretch as well (figure 1). This likely provokes a partial or total extrusion of the myristoyl group that is not induced by Ca^2+^, but rather by the presence of GRK1, which may facilitate the completion of the T to R transition that can occur with slightly decreased EC_50_ (table 2). Although the final protein-target complexes in the presence of Ca^2+^ have virtually indistinguishable tertiary (and quaternary) structures (figure 5, blue lines), intermediate conformational states are sampled which differ from one another both in terms of secondary (electronic supplementary material, figure S1) and tertiary (figure 5) structure.

In conclusion, we showed that Rec is able to sample a multitude of conformations depending on the presence of its multiple ligands: Ca^2+^ ions as well as the myristoyl moiety, that govern the state of fully soluble protein, and the GRK1 target as well as the disc membrane, which overall make mRec a peripheral membrane protein that can strongly interact with GRK1 target in a conformation that is different from its analogue in solution. By sampling these ligand-dependent conformations mRec ‘prepares' itself to promptly respond to the narrow drops in photoreceptor intracellular Ca^2+^ with a conformational transition that is highly cooperative, thus ensuring a rapid switching necessary for the fast events in phototransduction. In a broader perspective, our data support the notion that the regulation of Rh* phosphorylation by a Ca^2+^ feedback on GRK1 mediated by mRec is fully compatible with the Ca^2+^ dynamics occurring in higher vertebrates.

## 4. Methods

### 4.1. Protein expression and purification

Myristoylated Rec was heterologously expressed in *E. coli* BL21 (DE3) cells previously co-transformed with the plasmids encoding for Rec (pET11a-Rec) and N-myristoyl transferase (yNMT1) from *S. cerevisiae* (pBB131- yNMT1). Protein expression and purification were performed according to the protocols elucidated in [17,41] with slight modifications. Briefly, cell culture was grown at 37°C in LB medium containing 100 µg ml^−1^ ampicillin and 30 µg ml^−1^ kanamycin until OD_600_ reached 0.4, when the medium was added with 50 µg ml^−1^ myristic acid (in 50% EtOH) to allow myristoylation. Protein expression was induced at OD_600_ = 0.6 with 1 mM isopropyl-β-D-thiogalactopyranoside (IPTG). After 4 h at 37°C cells were harvested by centrifugation at 5000 × *g* for 15 min. Bacterial pellets were resuspended in 50 mM Tris–HCl pH 8 containing 1 mM MgCl_2_, 1X EDTA-free Protease Inhibitor Cocktail (Sigma), 0.1 mg ml^−1^ lysozyme and 5 U ml^−1^ DNAse I and incubated at 30°C for 30 min. After 30 min centrifugation at 20000 × *g* at 4°C, the supernatant was added with 0.5 mM CaCl_2_ and loaded onto an HiPrep Phenyl HP 16/10 (GE Healthcare) column previously equilibrated with 50 mM Tris–HCl pH 7.5, 0.5 mM CaCl_2_, 1 mM DTT, then eluted with 50 mM Tris–HCl pH 7.5, 2 mM EGTA, 1 mM DTT. Fractions purity was checked by SDS-PAGE. Rec was dialyzed against decalcified NH_4_HCO_3_, aliquoted after measuring the concentration by Bradford assay [42], lyophilized and stored at −80°C. Nonmyristoylated Rec was expressed and purified with the same procedure as for mRec, with the only difference being the absence of myristic acid and kanamycin during bacterial growth.

The peptide encompassing the first 25 amino acids of GRK1 (MDFGSLETVVANSAFIAARGSFDAS [10], hereafter ‘GRK1 peptide’), added with a neutral electric charge C-terminal tag (DGKGDK) for increased solubility [43] was purchased from GenScript, with a 97.1% purity, as assessed by HPLC. Peptide mass was estimated by amino acid analysis following acid hydrolysis.

### 4.2. Ca^2+^-binding assays

#### 4.2.1. Absorption competition assay using Br_2_-BAPTA

The Ca^2+^ affinity of Rec variants was estimated using two different methods based on the competition with a chromophoric chelator [44] as previously described [45]. The first method was based on monitoring the decrease in absorbance at *λ* = 263 nm of 28 µM 5,5′Br_2_-BAPTA (Ca^2+^ affinity = 2.3 µM) in the presence of 28 µM Rec upon addition of 3 µM Ca^2+^ for each titration point. Decalcified lyophilized proteins were solubilized in decalcified 20 mM Tris–HCl pH 7.5, 150 mM KCl buffer (initial [Ca^2+^]: 70 nM), protein concentration was calculated by Bradford assay [42], temperature was set at 25°C. In all experiments with the GRK1 peptide, the stoichiometric ratio Rec : GRK1 peptide was set to 1 : 1.5. Presented data were normalized as follows:

$$\text{normalized}\;A=\;\frac{A_{263}-A_{\min}}{A_{\max}-A_{\min}},$$

where *A*_263_ is the absorbance of each titration point, *A*_min_ and *A*_max_ are the absorbance values at the lowest and highest Ca^2+^ concentration, respectively.

#### 4.2.2. Fluorescence competition assay using Oregon Green BAPTA-5N

The second method was based on monitoring the fluorescence emission increase of 0.5 µM Oregon Green-conjugated BAPTA-5N (hereafter ‘OG BAPTA-5N’). The dye has a relatively low affinity for Ca^2+^ (20 µM at 22°C in 100 mM KCl, 10 mM MOPS, pH 7.2 according to Molecular Probes) and a maximum fluoresce emission at *λ* = 524 nm following excitation at 494 nm. Fifteen μM Rec was used in competition assays upon addition of 3 µM Ca^2+^ for the first 15 titration points followed by addition of 30 µM Ca^2+^ to verify Ca^2+^ saturation. Decalcified lyophilized proteins were dissolved in 10 mM MOPS pH 7.5, 100 mM KCl (initial [Ca^2+^]: 46–68 nM), temperature was set at 25°C, protein concentration was estimated by Bradford assay. Emission fluorescence spectra were recorded at 25°C between 500 and 560 nm after excitation at 494 nm in 1 cm quartz cuvettes, using a Jasco FP-750 spectrofluorometer, at a scan rate of 1 nm s^−1^, data pitch 2 nm. Data refer to an average of three accumulations subtracted with the spectrum of the buffer, and were normalized as follows

$$\text{normalized}\;F=1-\;\frac{F_{524}-F_{\min}}{F_{\max}-F_{\min}},$$

where *F*_524_ is the fluorescence emission of the dye at each titration point, *F*_min_ and *F*_max_ are the fluorescence values at the lowest and highest Ca^2+^ concentration, respectively. In all experiments with the GRK1 peptide, the stoichiometric ratio Rec : GRK1 peptide was set to 1 : 1.5.

The normalized signal decrease upon Ca^2+^ titration was fitted using CaLigator [44] to estimate individual macroscopic binding constants (*K*_d_i, table 1) to a two-sites binding model for nmRec and to a one-site binding model for mRec.

### 4.3. Preparation of liposomes

A mixture of lipids of the same composition of bovine ROS membranes [21] consisting of phosphatidylcholine, phosphatidylethanolamine, phosphatidylserine and cholesterol in a molar ratio of 40 : 40 : 15 : 5, was dissolved in chloroform and dried in a speed-vac concentrator. Lipid films (1 mg) were hydrated with 1 ml of 50 mM Tris–HCl pH 7.5, 150 mM KCl), vortexed for 30 min, sonicated for 15 min on ice, finally extruded 20 times through a 50 nm polycarbonate filter (Whatman). All lipids were purchased from Sigma Aldrich.

### 4.4. Dynamic light scattering measurements

Liposome (LP) size was assessed by dynamic light scattering (DLS) measurements using a Zetasizer Nano-S instrument (Malvern Instruments) with the same experimental settings as in [23], briefly consisting of water viscosity: 0.8872 cP, water refractive index: 1.33, liposome refractive index: 1.345, temperature: 25°C, equilibration time: 2 min, measurement angle: 173° backscatter, analysis model: multiple narrow modes. Data shown in electronic supplementary material, figure S2A represents an average of 12 measurements, each consisting of 13 repetitions. Polydispersion index and liposome size are reported as mean ± standard error.

### 4.5. Nanoparticle tracking analysis

Liposome size and concentration were estimated by Nanoparticle Tracking Analysis using a NanoSight instrument (Malvern), by acquiring three 1-min videos, each consisting of 25 frames s^−1^ at 25°C. Additional parameters: flow rate 20 µl min^−1^, camera level 16, detect threshold 5. Liposome size is reported as the mean of the mode in each of the three independent measurements ± standard error, liposome concentration is reported as mean ± standard error, data shown in electronic supplementary material, figure S2B refers to the average of three independent measurements.

### 4.6. Estimation of the stoichiometry of Rec-LP binding

The maximum number of Rec (*N*_Rec_) bound to LP was calculated according to the same geometrical model as explained in [23], based on the ratio between the area of the projection of a sphere representing Rec on the surface of a sphere representing LP

$$N_{\mathrm{Rec}}=\frac{4\pi {\left(r_{\mathrm{LP}}+r_{\mathrm{Rec}}\right)}^{2}}{\pi r_{\mathrm{Rec}}^{2}},$$

where *r*_LP_ is the radius of LP (37.65 nm assessed by nanoparticle tracking analysis) and *r*_Rec_ is the radius of Rec (2.2 nm). Under such assumptions, the maximum number of Rec molecules bound to a 75.3 nm LP is approximately 1400.

### 4.7. 8-Anilino-1-naphthalenesulfonic acid-based fluorescence titrations

An indirect method to assess the Ca^2+^ concentration at which half of the protein pool undergoes the T to R transition (EC_50_) consists of monitoring the conformational changes upon Ca^2+^ binding, which can be achieved by exploiting the fluorescence resonance energy transfer (FRET) phenomenon between fluorescence donors and acceptors that become very close to each-other upon interaction. The intrinsic fluorescence emission of Rec following excitation at 280 nm is essentially due to the three Trp residues (figure 1), whose emission band is centred around 340 nm. This fluorescence can therefore serve as donor for exciting the fluorescence of 8-Anilino-1-naphthalenesulfonic acid (ANS) when the probe binds to the hydrophobic patches of the protein. The maximal wavelength excitation for ANS in solution is indeed 380 nm, while maximal emission depends on the chemical environment, being 540 nm in the absence of hydrophobic interactors and shifting to 470 nm when ANS binds hydrophobic surfaces.

The emission fluorescence spectra were recorded between 300 and 550 nm at 25°C in 1 cm quartz cuvettes using a Jasco FP-750 spectrofluorometer, after excitation at 280 nm, at a scan rate of 1 nm s^−1^. Decalcified lyophilized proteins were dissolved in 80 mM HEPES pH 7.5, 40 mM KCl, 1 mM MgCl_2_, 25 µM ANS (initial [Ca^2+^]: 46–68 nM) to a final concentration of 2 µM Rec estimated by Bradford assay. In all experiments with the GRK1 peptide, the stoichiometric ratio Rec : GRK1 peptide was set to 1 : 1.5. Free Ca^2+^ concentration for each titration point, spanning from 3 nM to 800 µM, was obtained using Ca^2+^-EGTA buffer solution as previously explained [46,47]. Data refer to an average of three accumulations subtracted with the spectrum of the buffer. To minimize experimental differences between samples, the monitored signal is the ratio between the maximal intrinsic fluorescence emission (300–450 nm range) and the maximal ANS fluorescence emission (450–545 nm range), normalized as follows:

$$\text{normalized}\;I=\;\frac{I\;-I_{\min}}{I_{\max}-I_{\min}},$$

where *I* is the fluorescence ratio of each titration point, *I*_min_ and *I*_max_ are the fluorescence ratios at the lowest and highest Ca^2+^ concentration, respectively.

### 4.8. Circular dichroism spectroscopy and Ca^2+^ titrations

The second indirect method to evaluate the EC_50_, consisted in monitoring changes in secondary and tertiary structure of Rec upon Ca^2+^ titrations.

CD spectra were recorded essentially with the same methodology described in [48] using a Jasco J-710 spectropolarimeter supplied with a Peltier cell holder set at 25°C. Near UV spectra were recorded between 250 and 320 nm using a 1 cm quartz cuvette, while far UV spectra were collected between 200 and 250 nm using a 0.1 cm quartz cuvette. The free Ca^2+^ concentration for each titration point (between 420 pM and 1 mM) was calculated by MaxChelator (maxchelator.stanford.edu/CaEGTA-NIST.htm) using Ca-EGTA NIST database, additional parameters were temperature: 25°C, pH 7.5, ionic strength 0.15 M, EGTA 300 µM.

The spectrum of the solvent (50 mM Tris–HCl pH 7.5, 150 mM KCl) was considered as blank and subtracted; for the experiments with LP, the spectrum of 5 nM LP was considered as blank. Each spectrum represents five accumulations collected by setting scan rate: 50 nm min^−1^, bandwidth: 1 nm, response time: 4 s. Rec concentration in far UV spectra was adjusted to 10 µM, while for near UV spectra was set to 43 µM in the absence of LP and 7 µM in the presence of LP, since the LP suspension generated a prominent scattering of the incident light resulting in low signal-to-noise ratio, which prevented the collection of spectra at higher protein concentrations. In all experiments with the GRK1 peptide, the stoichiometric ratio Rec : GRK1 peptide was set to 1 : 1.5. Reported data refer to the average ± standard deviation after the following normalization:

$$\text{normalized }\;\theta_{\lambda }=\;\frac{\theta_{\lambda }-\theta_{\min}}{\theta_{\max}-\theta_{\min}},$$

where *θ_λ_* is the ellipticity of each titration point at fixed *λ* (279 nm for mRec and 292 nm for nmRec, where the largest spectral variations occurred, respectively), *θ*_min_ and *θ*_max_ are the ellipticity values at the lowest and highest Ca^2+^ concentration, respectively.

## Supplementary Material

Figures S1 - S3
