# Supplementary material for: Bringing the Ca2+ sensitivity of myristoylated recoverin into the physiological range
Source: Open Biol. 2021 Jan 6;11(1):200346. doi: 10.1098/rsob.200346 (PMC7881174; doi:10.1098/rsob.200346)
Supplement: Figures S1 - S3 [file rsob200346supp1.pdf]

Bringing the Ca^{2+}-sensitivity of myristoylated recoverin into the physiological range

by Valerio Marino, Matteo Riva[1], Davide Zamboni, Karl-Wilhelm Koch and Daniele Dell'Orco

Supplementary Material

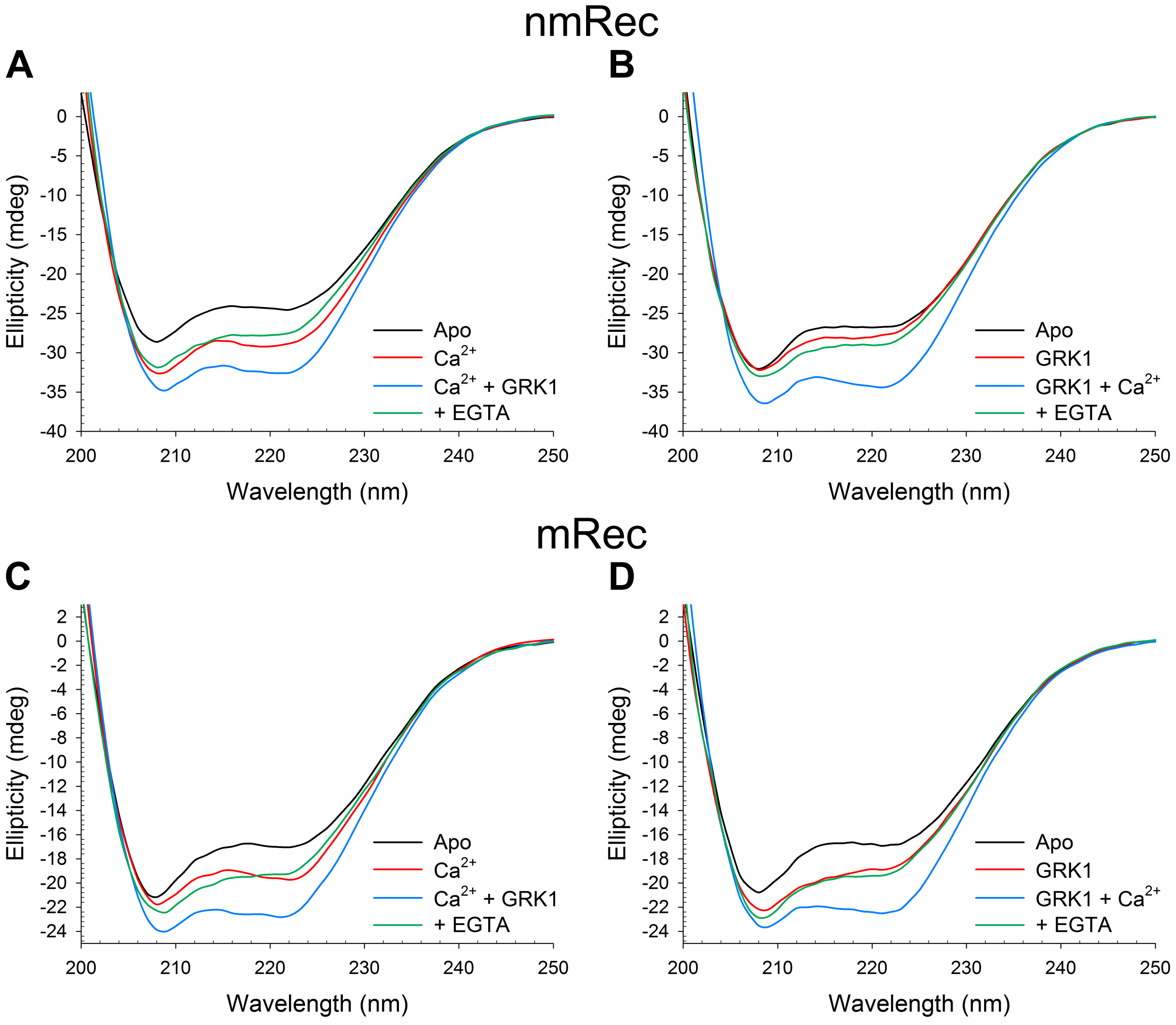

Figure S1. A) Far UV CD spectra of 10 μM nmRec and 300 μM EGTA (black), after sequential additions of 1 mM free Ca^{2+} (red), 15 μM GRK1 peptide (blue) and 4.2 mM free EGTA (green). B) Far UV CD spectra of 10 μM nmRec and 300 μM EGTA (black), after sequential additions of 15 μM GRK1 peptide (red), 1 mM free Ca^{2+} (blue) and 4.2 mM free EGTA (green). C) Far UV CD spectra of 10 μM mRec and 300 μM EGTA (black), after sequential additions of 1 mM free Ca^{2+} (red), 15 μM GRK1 peptide (blue) and 4.2 mM free EGTA (green). D) Far UV CD spectra of 10 μM mRec and 300 μM EGTA (black), after sequential additions of 15 μM GRK1 peptide (red), 1 mM free Ca^{2+} (blue) and 4.2 mM free EGTA (green).

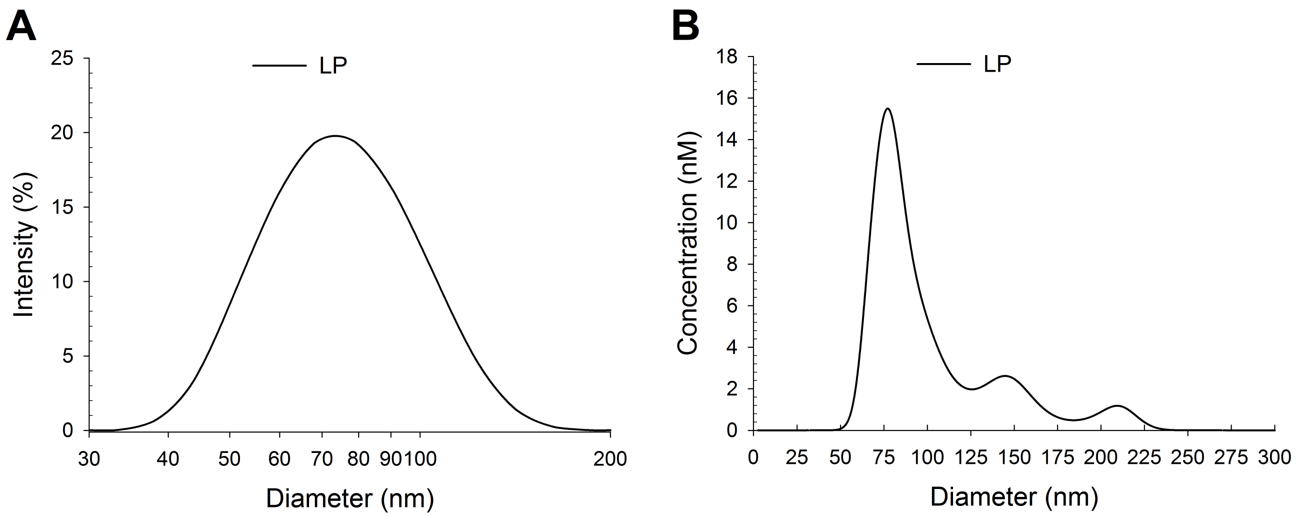

Figure S2. Hydrodynamic diameter estimation of 15 nM LP monitored by A) DLS and B) Nanoparticle Tracking Analysis.

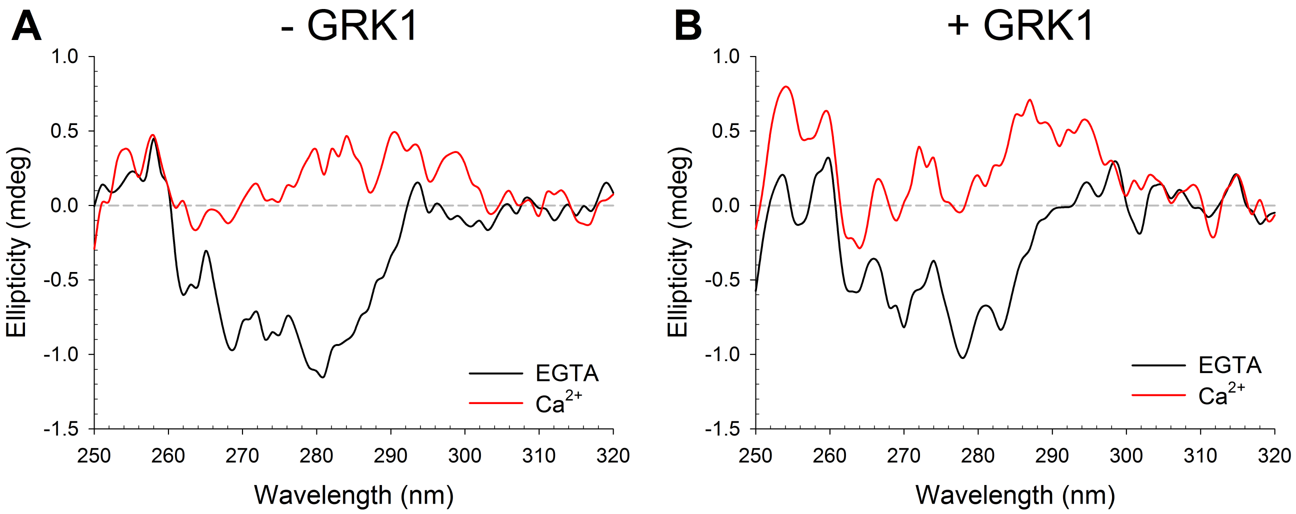

Figure S3. Near UV CD spectra of 7 µM mRec in the presence of 5 nM LP and 300 µM EGTA (black) or 1 mM Ca^{2+} (red). B) Near UV CD spectra of 7 µM mRec in the presence of 5 nM LP, 10.5 µM GRK1 peptide and 300 µM EGTA (black) or 1 mM Ca^{2+}.
